# Surveillance of noise exposure levels in workplaces in Beijing

**DOI:** 10.3389/fpubh.2025.1486497

**Published:** 2025-04-29

**Authors:** Yan Dong, Yuqian Li, Yan Liu, Yan Zhao, Heng Zhang, Yun Sun, Zizi Li

**Affiliations:** ^1^Institute of Urban Safety and Environmental Science, Beijing Academy of Science and Technology, Beijing, China; ^2^National Institute for Nutrition and Health, Chinese Center for Disease Control and Prevention, Beijing, China

**Keywords:** surveillance, occupational noise exposure, workplace, risk factors, Beijing

## Abstract

Occupational noise-induced deafness is the second most prevalent occupational disease in China after pneumoconiosis. This study aims to estimate overall noise exposure levels and trends in industrial enterprises in Beijing. A total of 286 enterprises were monitored, and the median and interquartile range (IQR) of noise exposure levels were calculated. The distribution of noise exposure level was analyzed, revealing that 116 enterprises exceeded permissible noise limits. In terms of industry, noise exceedance was observed in 28 enterprises engaged in metal product manufacturing (24.13%) and 27 enterprises in motor vehicle manufacturing (23.28%). Regarding ownership type, 71 limited liability companies exhibited noise exceedance, making up 61.20% of the total. Multivariate logistic regression identified industry-specific risk factors associated with noise exceedance. The risk was significantly higher in metal product manufacturing (OR = 3.31, 95% CI: 1.61–6.82) and railway, shipping, aerospace, and other transportation equipment manufacturing (OR = 12.18, 95% CI: 1.40–105.63). Conversely, enterprises engaged in printing and recorded media reproduction exhibited a lower risk (OR = 0.29, 95% CI: 0.13–0.67). The noise exceedance rate in Beijing is lower than the national average, and levels in line closely with nationwide standards. Detailed surveillance of noise exposure levels provides a basis for occupational health authorities to conduct targeted supervision and formulate industry-specific control measures for high-risk sectors and job roles.

## 1 Introduction

By 2050, nearly 2.5 billion people are projected to have some level of hearing loss. It has been found that exposure to loud noise or sounds and ototoxic chemicals in the workplace are crucial risk factors throughout life (1). Worldwide, 16% of the disabling hearing loss in adults (over 4 million DALYs) is attributed to occupational noise as of 2000 (2). In 2019, the global age-standardized disability-adjusted life year rate for occupational noise-induced hearing loss was recorded at 84.23 per 100,000 population (3). Besides, occupational noise exposure is associated with non-auditory diseases, such as hypertension, metabolic syndrome, electrocardiograph abnormality, hyperglycaemia and insomnia (4–8). A systematic review and meta-analysis of cohort studies found that living or working in environments with noise exposure was significantly associated with an increased risk of hypertension (RR = 1.18, 95% CI: 1.06–1.32). Additionally, for every 10 dB(A) increase in noise exposure, the summary risk ratio for hypertension was 1.13 (95% CI: 0.99 to 1.28) (9). A 15-year follow-up of a nationwide prospective cohort in Switzerland demonstrated that exposure to road traffic, railway and aircraft noise was associated with cardiovascular disease (CVD) mortality, with adjusted hazard ratios (95% CI) of 1.029 (1.024–1.034), 1.013 (1.010–1.017), and 1.003 (0.996–1.010) per 10 dB Lden, respectively (10). The combined impact of auditory and non-auditory diseases due to noise exposure is expected to impose a significant medical burden.

In China, from 2017 to 2022, the proportion of otolaryngology and oral diseases among newly diagnosed occupational diseases increased from 6.01 to 16.91% (11, 12), with an annual incidence growth rate of 24.2% since 2014. Occupational noise-induced deafness has been the second leading occupational disease in China after pneumoconiosis (13). Research by Bai indicates a steady increase in occupational otolaryngology and oral diseases in Beijing from 2005 to 2021, with the vast majority being noise-induced deafness, primarily observed in the manufacturing industry (14). Li’s study reported that the rates of occupational noise exceedance were 13.6, 13.8, 17.4, and 16.9% 13.6, 13.8, 17.4 and 16.9% in industries such as printing and reproduction of recorded media industry, manufacturing of motor vehicles, computers, communications, and other electronic equipment manufacturing, as well as manufacturing of metal products. Further analysis revealed that, compared to large enterprises, medium-sized enterprises (*p* = 0.001, OR = 6.6, 95% CI: 2.5–17.7), small enterprises (*p* < 0.001, OR = 47.3, 95% CI: 15.2–147.6), and micro enterprises (*p* < 0.001, OR = 15.0, 95% CI: 4.4–50.7) faced significantly higher risks of excessive noise exposure (15, 16). However, previous studies have primarily focused on specific industries and have not provided comprehensive and up-to-date information on noise exposure in manufacturing enterprises in Beijing.

Since 2014, Beijing has initiated the relocation of non-capital functions and traditional manufacturing industries, such as mining, to reduce the number of enterprises with high pollution, high energy consumption and high water consumption. This restructuring has altered the distribution of manufacturing industries in Beijing (15, 16). However, its impact on overall noise levels in the manufacturing sector remains unknown. It is necessary to understand the status quo of noise hazards across various industries in Beijing. It is crucial to analyze noise distribution and intensity in workplaces of varying sizes and ownership types to provide a scientific basis for regulatory supervision, law enforcement, and the research and revision of occupational disease prevention and control regulations, standards, and guidelines.

## 2 Methods

### 2.1 Subjects

To estimate the overall noise exposure level and trends in industrial enterprises in Beijing, a citywide monitoring initiative was implemented. Selected enterprises met the requirements outlined in the *Work Plan for Surveillance of Occupational Hazards in the Workplace* (2023), issued by the National Health Commission of the People’s Republic of China. The *Work Plan* specified the scope of industries, job positions and occupational hazard factors subject to surveillance. According to a preliminary citywide survey, 286 enterprises were included in the 2023 noise surveillance program. These enterprises represented all entities in Beijing that met the eligibility criteria specified in the monitoring program. Surveillance was primarily conducted by county-level Occupational Disease Prevention and Control Institutes, along with occupational health technical service institutions. The selected enterprises, from 16 districts in Beijing, were categorized based on their industrial classification, enterprise scale, and ownership type (17–19).

According to the enterprise scale, the measurement quantity requirements were required to select: (i) for large and medium-sized enterprises, each employer was required to select no fewer than four noise-exposed posts for monitoring. Noise intensity was measured at all workplaces associated with these posts; and (ii) for small and micro-sized enterprises, all posts involving noise exposure were to be measured. Quality control of the surveillance was overseen by the Institute of Urban Safety and Environmental Science, Beijing Academy of Science and Technology, in accordance with the *Work Plan for Surveillance of Occupational Hazards in the Workplace (2023)*.

### 2.2 Measurements of A-weighted equivalent continuous sound pressure level

According to the standard *Measurement of Noise in the Workplace (GBZ/T 189.8–2007)* (20), the measuring instruments used include sound level meters, integrating sound level meters, or personal noise dosimeters. All measuring instruments must be Type 2 or higher and equipped with A-weighting and S (slow) settings. Additionally, integrating sound level meters or personal noise dosimeters must have a “Peak” setting. When selecting measuring instruments, a sound level meter is used for fixed workstations, while a personal noise dosimeter is prioritized for mobile workstations. Alternatively, a sound level meter can be used at different work locations, and the equivalent sound level calculated accordingly. The measurement follows the specified Equation 1.


(1) $$L_{Aeq,T}=10\mathrm{lg}\;\left(\frac{1}{T}\overset{n}{\underset{i=1}{\sum }}Ti10^{0.1\left(\mathrm{LAeq,Ti}\right)}\right)\left[\mathrm{dB}\left(A\right)\right]$$

Where L_Aeq,T_ is the equivalent sound level throughout the workday, L_Aeq,Ti_ is the equivalent sound level during a specific time period T_i_, T is the total duration of all time periods, T_i_ is the duration of the time period T_i_, and n is the total number of time periods.

The integrating sound level meter or personal noise dosimeter must be configured with A-weighting and S (slow) time weighting, with measurements recorded as either the A-weighted sound level (L_PA_) or A-weighted equivalent continuous sound pressure level (L_Aeq_). During measurement, the microphone should be positioned at the worker’s ear height and oriented toward the sound source. The measuring instrument should be mounted on a tripod and placed at the designated measurement point. If tripod placement is not feasible, the sound level meter may be handheld, ensuring that the distance between the tester and the microphone remains >0.5 m. Principles for selecting measurement points are as follows: (i) If the A-weighted sound level difference within the measurement range is <3 dB(A), three measurement points should be selected, and the average value should be taken; (ii) If the sound field is unevenly distributed, it should be divided into multiple zones, each with a sound level difference of <3 dB(A). Two measurement points should be selected in each zone, and the average value should be calculated; and (iii) noise levels should be measured separately at each work location within the worker’s range of movement, and the equivalent sound level should be calculated. For impulsive noise measurement, the “Peak” detection setting should be employed. In workplaces with steady-state noise, three consecutive measurements shall be conducted at each monitoring point, with the average value calculated. In non-steady-state noise environments, measurement periods should be divided based on sound level fluctuations (≥3 dB variation), with equivalent sound levels recorded for each period along with their respective durations. When assessing impulsive noise, both the peak sound pressure level and the cumulative number of impulsive events during the work shift must be quantified.

### 2.3 Calculation of noise exposure levels

In this study, noise exposure levels were assessed as time-weighted average exposures over specified periods and expressed as L_Ex, 8h_/L_Ex, 40h_. L_Ex, 8h_/L_Ex, 40h_. These values were calculated based on the equivalent continuous sound level and exposure duration (8 h or 40 h) across all monitored workplaces, following *Measurement of noise in the workplace* (*GBZ/T 189.8–2007*) (20). L_Ex, 8h_ is an average exposure weighted to account for time and changing noise levels over an 8-h workday, while L_Ex, 40h_ reflects the exposure over a 40-h workweek. The measurement follows the specified Equations 2, 3.


(2) $$L_{\mathrm{Ex},8h}=L_{Aeq,Te}+10\mathrm{lg}\left(T_{e}/T_{0}\right)\left[\mathrm{dB}\left(A\right)\right]$$

(3) $$L_{\mathrm{Ex},40h}=10\mathrm{lg}\;\left(\frac{1}{5}\overset{n}{\underset{i=1}{\sum }}10^{0.1\left(L_{\mathrm{Ex},8h}\right)}i\right)\left[\mathrm{dB}\left(A\right)\right]$$

Where L_Aeq,Te_ is the equivalent sound level for the actual workdays, T_e_ is actual working hours, T_0_ is standard working hours (8-h), and n is the number of events in time T.

### 2.4 Determination of noise exposure exceedance

*Occupational Exposure Limits for Hazardous Agents in the Workplace, Part 2: Physical Agents* (*GBZ 2.2–2007*) is a mandatory national occupational health standard issued by the National Health Commission of the People’s Republic of China. This standard defines the exposure limits of physical agents in the workplace, including noise, ultrahigh frequency radiation, high-frequency electromagnetic fields, laser radiation, microwave radiation, ultraviolet radiation, heat stress work, and hand-transmitted vibration, among others. The noise exposure limits are based on *GBZ 2.2–2007* (21). The occupational exposure limits for noise are as follows: (i) for a 5-day workweek with 8 working hours per day, the steady-state noise limit is 85 dB(A), and the equivalent sound level limit for unsteady-state noise is also 85 dB(A); (ii) For a 5-day workweek with non-8-h workdays, the 8-h equivalent sound level should be calculated, with a limit of 85 dB(A); and (iii) if the workweek is not 5 days, the equivalent sound level for a 40-h workweek must be calculated, with a limit of 85 dB(A). In this study, noise exceedance was defined as L_Ex, 8h_/L_Ex, 40h_ ≥ 85 dB(A). In any monitoring post in an enterprise exceeded 85 dB(A), the enterprise was classified as having a noise exceedance.

### 2.5 Statistical analysis

After normality test analysis, the L_Ex, 8h_/L_Ex, 40h_ was found to be non-normally distributed. The median and interquartile range (IQR) were calculated to describe the distribution of continuous variables with non-normally distributed data. The Kruskal-Wallis H test was employed to compare the differences in non-normal distributions between groups. The chi-square test was used to compare noise excess rates between the two groups. When the number of cases was <40 or the theoretical number was <1, Fisher’s exact probability test was used to calculate the exact *p*-value. Cases where Fisher’s exact test was not feasible due to insufficient computer memory, a Monte Carlo simulation (with n set to 500,000 in the PROC FREQ procedure) was used to estimate the exact p-value. In this study, the independent variable, noise exposure level, was treated as either an ordinal or continuous variable. The presence or absence of earplugs provided and earplugs worn were treated as binary (yes or no). The Cochran Armitage test was conducted to examine the trend in the proportions of earplugs provided and earplugs worn with increasing noise exposure, with Z and *p* values calculated.

A multivariate logistic regression model with stepwise adjustment of variables was performed to determine factors associated with noise exceedance in enterprises under study. The results were presented as ORs with 95% confidence intervals (CIs). These variables included in the model were enterprise scale (large and medium, small and micro-sized), district, operating years, participation of managers in occupational health training (yes or no), participation of occupational health managers in occupational health training (yes or no), provision of earplugs (yes or no), posting of noise warning signs, utilization of noise reduction devices (yes or no), divisions, and ownership type of enterprises. The assignment of variables is shown in Supplementary Table 1. Correlations between the districts and divisions for enterprises in this study were evaluated by Spearman rank correlation analyses, with a heatmap used to visualize these correlations. Statistical analysis was conducted using SAS 9.4 (SAS Institute Inc., Cary, NC, USA). Figures were processed using R Studio (version 4.3.2), with R packages, including Magritte, dplyr, forest plot and ggplot 2, used to process the figures. The significance level for all tests was set at *p* < 0.05 (two-sided).

## 3 Results

### 3.1 Basic information

Table 1 presents the basic information about the enterprises included in this study. A total of 286 enterprises were included: 47 large and medium-sized enterprises and 239 small and micro-sized enterprises. The median operating duration was 19.5 years. Both managers and occupational health managers from 92.66% of the enterprises participated in occupational health training. A total of 252 enterprises (88.11%) posted noise warning signs, while 143 enterprises (50%) used noise reduction facilities. Additionally, 282 enterprises (98.6%) provided earplugs, and 242 enterprises (84.62%) ensured that all employees wore earplugs. Statistical analysis indicated significant differences (*p* < 0.05) in the characteristics between large and medium-sized enterprises and small and micro-sized enterprises.

**Table 1:** Characteristics of enterprises in this study.

Stratification variables	Large and medium enterprises	Small and micro-sized enterprises	Total	Statistic	
				*χ^2^*/F	*P*
Operating years of enterprises, years	20.0 (13.0, 26.0)	19.0 (14.0, 27.0)	19.5 (14.0, 27.0)	0.3724	0.5417
Proportions of managers participating in occupational health training, *n* (%)	44 (93.62)	221 (92.47)	265 (92.66)	0.7045	1.0000
Proportions of occupational health managers participating in occupational health training, *n* (%)	44 (93.62)	221 (92.47)	265 (92.66)	0.7045	1.0000
Posting of noise warning signs, *n* (%)	44 (93.62)	208 (87.03)	252 (88.11)	1.6272	0.2021
Utilization of noise reduction facilities, *n* (%)	29 (61.70)	114 (47.70)	143 (50.00)	3.0807	0.0792
Proportions of earplugs provided, *n* (%)	46 (97.87)	236 (98.74)	282 (98.60)	0.5144	0.5144
Proportions of earplugs wearing, *n* (%)	41 (87.23)	201 (84.10)	242 (84.62)	0.7733	0.6652

Enterprises in this study were categorized by industry, ownership, and district. The distribution of enterprises was described by these factors and expressed as *n* and %: (i) in terms of district distribution, District H and District J had the highest number of enterprises, each with 40, while District A had the fewest, with only one enterprise (Supplementary Figure 1a); (ii) By industry, the manufacturing of motor vehicles had the highest number of enterprises, with 57, whereas the electricity and heat production and supply industry, ferrous metal smelting and rolling industry, and manufacturing of coke and refined petroleum products each had only one enterprise (Supplementary Figure 1b); as for ownership, limited liability companies dominated, with 174 enterprises, while joint-operated companies had the fewest, with only two (Supplementary Figure 1c). Other results are shown in Supplementary Figures 1a–c.

**Figure 1:**
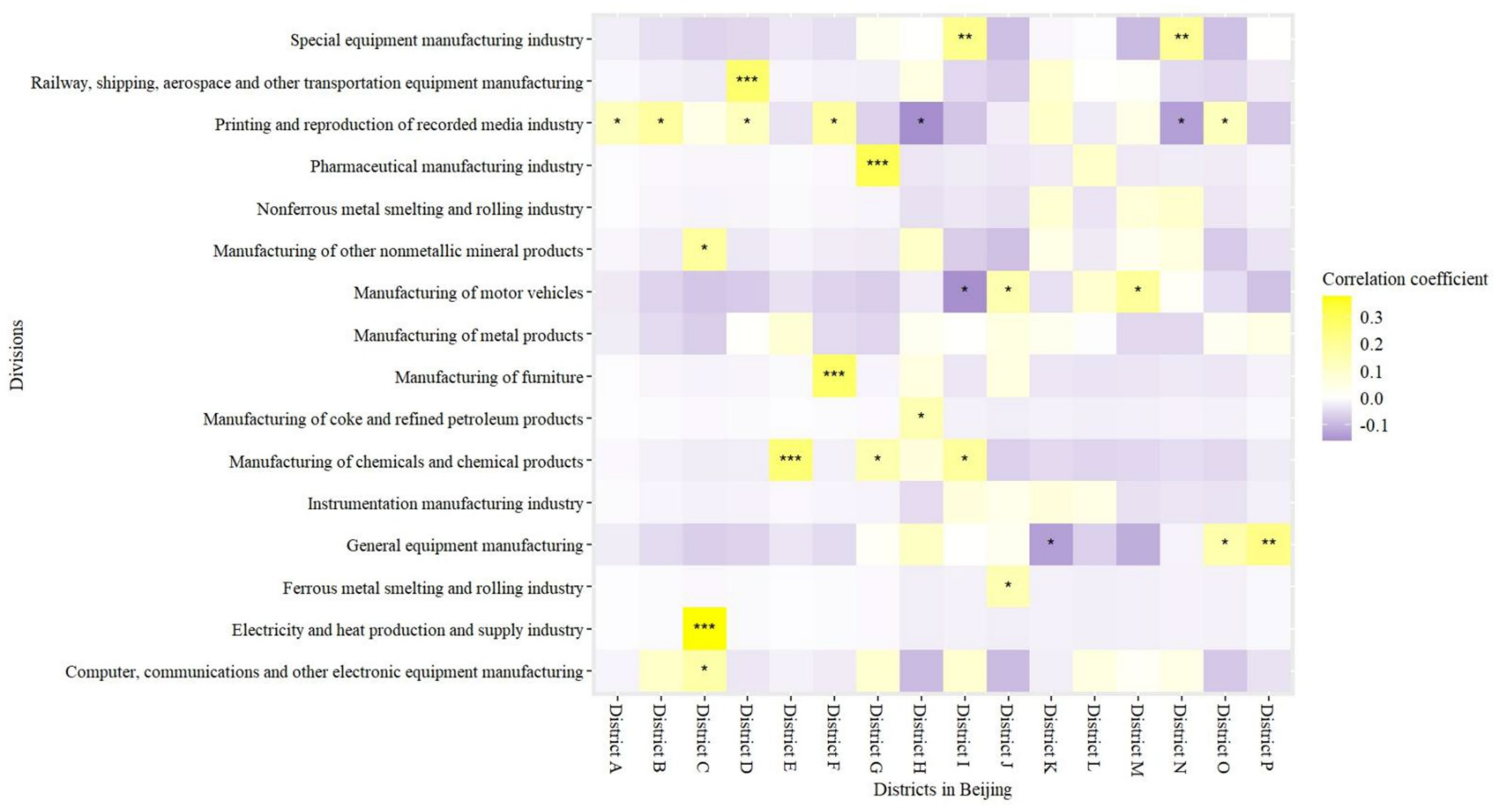
Heatmap for the correlations between different industries and districts in Beijing using Spearman correlation analysis. *p < 0.05, **p < 0.01, ***p < 0.001.

The correlations between different industries and districts in Beijing were visualized using a heatmap (Figure 1), with colors indicating positive correlation (yellow) or negative correlation (blue). Figure 1 reveals clustering in the distribution of industries in different districts in Beijing. Specifically, the main industries in District J are the ferrous metal smelting and rolling industry and the manufacturing of motor vehicles, while District G is characterized by the manufacturing of chemicals and chemical products, as well as pharmaceutical manufacturing. Other results are shown in Figure 1.

### 3.2 Noise exposure analysis

Table 2 indicates the levels of noise exposure in measuring positions with different characteristics in this study. The median and interquartile range (IQR) were used to describe noise exposure levels. The proportion of noise exposure levels ≥ 85 dB(A) (PINE ≥ 85) is shown in both number and percentage. Data from 1,690 posts were included, indicating an overall PINE ≥ 85 of 21.89% and an overall noise exposure level of 81.1(77.6, 84.3) dB(A): (i) for enterprises in different districts, those in District J showed the highest noise exposure, while those in District A exhibited the lowest. The highest PINE ≥ 85 was observed in District K, while the lowest was in District A, District B, and District G; (ii) in terms of industry, the furniture manufacturing sector exhibited the highest noise exposure and PINE ≥ 85, while the chemical manufacturing sector had the lowest noise exposure levels. The coke and refined petroleum products manufacturing, as well as the pharmaceutical manufacturing industries, showed the lowest PINE ≥ 85. Large and medium-sized enterprises generally had slightly higher noise exposure and PINE ≥ 85 compared to small and micro-sized ones; and (iii) Regarding ownership types, private enterprises exhibited the lowest noise exposure, while enterprises from Hong Kong, Macao, and Taiwan had the highest. Joint-equity cooperative enterprises had the lowest PINE ≥ 85, while those invested by Hong Kong, Macao, and Taiwan exhibited the highest.

**Table 2:** Distribution of noise exposure levels monitoring posts among the industry in different stratification - Beijing, 2023.

Stratification variables	Number of samples, *n*	Noise exposure level L_Ex,8h_/L_Ex,40h_* [dB(A)]		
		*M* (*Q_1_*, *Q_3_*)	Min, Max	The proportion of noise exposure levels ≥85 dB(A) (*n*, %)
Districts				
District A	3	72.3 (70.0, 72.4)	70.0, 72.4	0 (0)
District B	9	74.2 (70.0, 77.5)	70.0, 79.9	0 (0)
District C	42	81.2 (78.6, 84.5)	71.1, 91.9	6 (14.29)
District D	22	80.2 (76.3, 81.3)	71.7, 103.9	2 (9.09)
District E	10	74.5 (70.3, 77.4)	70.3, 88.3	1 (10.00)
District F	15	78.6 (76.2, 86.4)	70.7, 90.7	4 (26.67)
District G	10	73.6 (71.0, 80.5)	70.3, 84.6	0 (0)
District H	198	80.7 (77.2, 83.5)	70.0, 97.8	35 (17.68)
District I	193	80.1 (76.8, 84.2)	70.0, 101.7	46 (23.83)
District J	367	82.5 (78.0, 85.9)	70.0, 105.1	117 (31.88)
District K	253	82.3 (78.9, 87.7)	70.4, 100.8	86 (33.99)
District L	175	81.1 (77.5, 84.1)	70.0, 101.1	33 (18.86)
District M	182	81.2 (78.3, 83.0)	70.70 89.6	2 (1.10)
District N	99	80.2 (74.8, 83.3)	70.0, 94.9	16 (16.16)
District O	83	81.8 (79.7, 84.7)	72.1, 100.7	19 (22.89)
District P	29	79.4 (76.2, 81.9)	71.0, 88.8	3 (10.34)
Statistic		*H* = 98.1407, *p* < 0.0001		*P* < 0.0001
Industries				
Electricity and heat production and supply industry	20	82.9 (79.3, 84.8)	77.1, 87.1	4 (20.00)
Manufacturing of other nonmetallic mineral products	134	80.7 (77.2, 83.8)	70.4, 100.6	26 (19.40)
Ferrous metal smelting and rolling industry	10	83.4 (80.6, 87.5)	73.2, 105.1	3 (30.00)
Manufacturing of chemicals and chemical products	31	76.2 (71.0, 79.5)	70.0, 87.6	2 (6.45)
Computer, communications and other electronic equipment manufacturing	67	79.7 (73.0, 82.6)	70.0, 101.1	9 (13.43)
Manufacturing of furniture	21	85.3 (80.6, 86.4)	71.9, 95.7	11 (52.38)
Manufacturing of metal products	292	81.5 (77.1, 85.7)	70.0, 97.8	81 (27.74)
Manufacturing of motor vehicles	318	82.0 (79.2, 85.0)	70.5, 95.7	79 (24.84)
Manufacturing of coke and refined petroleum products	3	79.3 (78.0, 82.7)	78.0, 82.7	0 (0.00)
Railway, shipping, aerospace and other transportation equipment manufacturing	92	83.5 (79.0, 87.8)	71.7, 103.9	35 (38.04)
General equipment manufacturing	188	80.1 (75.4, 83.9)	70.0, 101.7	40 (21.28)
Pharmaceutical manufacturing industry	6	83.4 (82.1, 84.3)	79.2, 84.6	0 (0.00)
Instrumentation manufacturing industry	16	81.0 (74.8, 87.2)	70.8, 100.8	4 (25.00)
Printing and reproduction of recorded media industry	259	81.2 (77.9, 83.1)	70.0, 93.7	26 (10.04)
Nonferrous metal smelting and rolling industry	25	81.6 (80.7, 83.6)	71.9, 95.2	3 (12.00)
Special equipment manufacturing industry	208	80.4 (76.7, 83.9)	70.0, 96.6	47 (22.60)
Statistic		*H* = 89.4629, *P* < 0.0001		*P* < 0.0001
Enterprise-scale				
Large and medium enterprises	380	81.4 (78.0, 85.9)	70.1, 105.1	105 (27.63)
Small and micro-sized enterprises	1,310	81.1 (77.5, 84.1)	70.0, 101.7	265 (20.23)
Statistic		*H* = 6.1861, *p* = 0.0129		*F* = 0.9990, *p* = 0.0030
Ownership type				
State-owned	204	81.4 (77.2, 84.7)	70.0, 103.9	47 (23.04)
Collective	39	80.4 (76.6, 82.7)	70.0, 93.7	2 (5.13)
Joint-equity cooperative enterprises	10	80.5 (77.2, 81.5)	70.0, 81.5	0 (0.00)
Joint-operate	10	79.4 (72.4, 82.9)	71.7, 87.1	2 (20.00)
Private	86	78.6 (76.0, 82.2)	70.0, 94.9	11 (12.79)
Incorporated company	27	80.6 (75.9, 87.9)	70.8, 100.8	8 (29.63)
Limited liability company	1,023	81.2 (77.9, 84.5)	70.0, 105.1	226 (22.09)
Hong Kong, Macau, and Taiwan invested enterprises	7	84.1 (80.9, 89.5)	79.4, 94.9	3 (42.86)
Foreign	214	81.5(78.2, 85.1)	70.3, 101.7	54 (25.23)
Others	70	80.7 (77.2, 85.0)	70.0, 101.1	17 (24.29)
Statistic		*H* = 26.4413, *p* = 0.0017		*p* = 0.0170
Total	1,690	81.1 (77.6, 84.4)	70.0, 105.1	370 (21.89)

*LEx,8h: Normalization of equivalent continuous A-weighted sound pressure level to a nominal 8 h working day. LEx,40h: Normalization of equivalent continuous A-weighted sound pressure level to a nominal 40 h working week.

### 3.3 Analysis of excessive noise

Table 3 presents the noise exceedance rates among enterprises with different characteristics. The overall noise exceedance rate for enterprises in Beijing was 40.56%, with large and medium-sized enterprises exhibiting a higher exceedance rate of 59.57%, compared to 36.82% for small and micro-sized enterprises. Across different districts, District J recorded the highest noise exceedance rate at 70%, with 28 out of 40 enterprises exceeding the limit. In contrast, Districts A, B, and G had the lowest exceedance rate at 0%. Regarding industrial divisions, the highest noise exceedance rates were observed in the electricity and heat production and supply industry, the ferrous metal smelting and rolling industry, and the furniture manufacturing industry, followed by the railway, shipping, aerospace, and other transportation equipment manufacturing sector. Conversely, the manufacturing of coke and refined petroleum products, along with the pharmaceutical manufacturing industry, exhibited the lowest noise exceedance rates. With respect to ownership types, foreign-owned enterprises and those classified under “Other” ownership had the highest noise exceedance rate at 55.56%, while joint-equity cooperative enterprises recorded the lowest rate.

**Table 3:** The ratio of noise exceedance among enterprises with different characteristics in Beijing, 2023.

Stratification variables	Large and medium enterprises	Small and micro-sized enterprises	Total	Statistic	
				*χ^2^*	*P*
District, *n* (%)					
District A	0 (0)	0 (0)	0 (0)	–	–
District B	0 (0)	0 (0)	0 (0)	–	–
District C	2 (100.00)	0 (0)	2 (28.57)	1.0000	0.0476
District D	1 (100.00)	1 (20.00)	2 (33.33)	1.0000	0.3333
District E	0 (0)	1 (100.00)	1 (50.00)	0.5000	1.0000
District F	0 (0)	1 (33.33)	1 (25.00)	0.7500	1.0000
District G	0 (0)	0 (0)	0 (0)	–	–
District H	3 (60.00)	11 (31.43)	14 (35.00)	0.9574	0.3223
District I	1 (50.00)	14 (58.33)	15 (57.69)	0.6769	1.0000
District J	7 (70.00)	21 (70.00)	28 (70.00)	0.6447	1.0000
District K	5 (83.33)	8 (38.10)	13 (48.15)	0.9942	0.0768
District L	5 (62.50)	12 (46.15)	17 (50.00)	0.8877	0.6880
District M	1 (25.00)	0 (0)	1 (3.23)	1.0000	0.1290
District N	3 (75.00)	6 (31.58)	9 (39.13)	0.9858	0.2601
District O	0 (0)	11 (40.74)	11 (39.29)	0.6071	1.0000
District P	0 (0)	2 (25.00)	2 (25.00)	–	–
Industries, *n* (%)					
Electricity and heat production and supply industry	1 (100.00)	0 (0)	1 (100.00)	–	–
Manufacturing of other nonmetallic mineral products	2 (66.67)	2 (20.00)	4 (30.77)	0.9860	0.2028
Ferrous metal smelting and rolling industry	1 (100.00)	0 (0)	1 (100.00)	–	–
Manufacturing of chemicals and chemical products	0 (0)	2 (33.33)	2 (28.57)	0.7143	1.0000
Computer, communications and other electronic equipment manufacturing	3 (50.00)	2 (22.22)	5 (33.33)	0.9530	0.3287
Manufacturing of furniture	0 (0)	3 (100.00)	3 (100.00)	–	–
Manufacturing of metal products	0 (0)	28 (66.67)	28 (65.12)	0.3488	0.3488
Manufacturing of motor vehicles	13 (72.22)	14 (35.90)	27 (47.37)	6.5183	0.0107
Manufacturing of coke and refined petroleum products	0 (0)	0 (0)	0 (0)	–	–
Railway, shipping, aerospace and other transportation equipment manufacturing	2 (100.00)	5 (83.33)	7 (87.50)	1.0000	1.0000
General equipment manufacturing	0 (0)	12 (29.27)	12 (27.91)	0.5150	1.0000
Pharmaceutical manufacturing industry	0 (0)	0 (0)	0 (0)	–	–
Instrumentation manufacturing industry	1 (100.00)	1 (33.33)	2 (50.00)	1.0000	1.0000
Printing and reproduction of recorded media industry	0 (0)	8 (17.02)	8 (16.00)	0.5857	1.0000
Nonferrous metal smelting and rolling industry	0 (0)	2 (66.67)	2 (66.67)	–	–
Special equipment manufacturing industry	5 (71.43)	9 (32.14)	14 (40.00)	0.9901	0.0897
Ownership type, *n* (%)					
State-owned	6 (54.55)	5 (20.83)	11 (31.43)	–	0.5691
Collective	0 (0)	2 (25.00)	2 (25.00)		
Joint-equity cooperative enterprises	0 (0)	0 (0)	0 (0)		
Joint-operate	1 (100.00)	0 (0)	1 (50.00)		
Private	0 (0)	7 (36.84)	7 (36.84)		
Incorporated company	1 (50.00)	1 (33.33)	2 (40.00)		
Limited liability company	13 (59.09)	58 (38.16)	71 (40.80)		
Hong Kong, Macau, and Taiwan invested enterprises	0 (0)	2 (50.00)	2 (50.00)		
Foreign	5 (62.50)	10 (52.63)	15(55.56)		
Others	2 (66.67)	3 (50.00)	5 (55.56)		
Total	28 (59.57)	88 (36.82)	116 (40.56)	8.4350	0.0037

Table 3 shows that a total of 116 enterprises experienced noise exceedance. By district, 28 enterprises in District J exceeded noise limits, accounting for 24.13% of the total. In terms of industry, 28 enterprises in the metal products manufacturing sector and 27 enterprises in the motor vehicle manufacturing sector reported noise exceedance, representing 24.13 and 23.28% of the total, respectively. Regarding ownership type, 71 limited liability companies experienced noise exceedance, comprising 61.20% of all affected enterprises.

**Figure 2:**
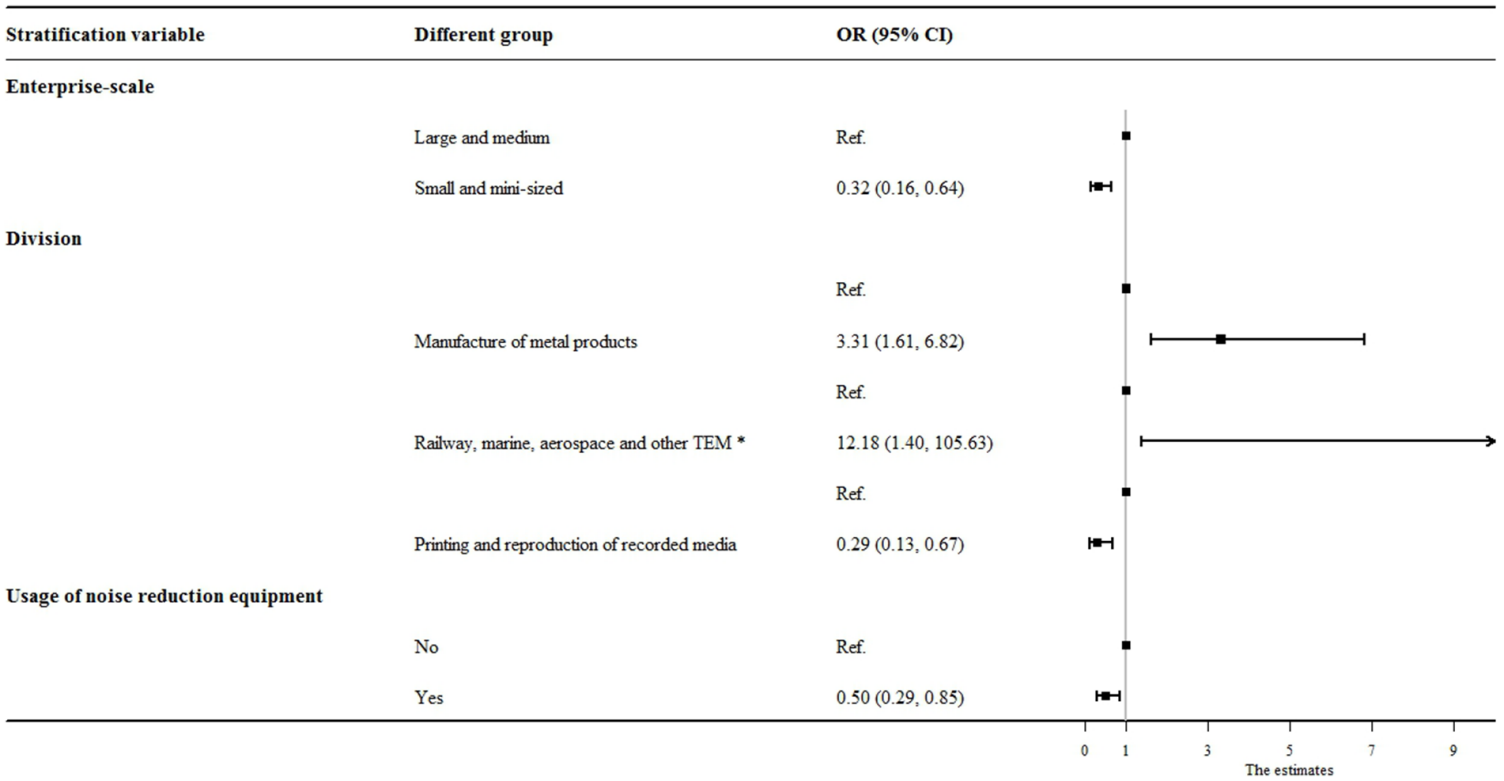
Influential factors associated with noise exceedance in industries within this study. *TEM was the abbreviation for transportation equipment manufacturing.

A multivariate logistic regression model with stepwise adjustment of variables was performed to assess factors associated with noise exceedance in enterprises under study. Through stepwise regression analysis, factors such as enterprise scale, industry, and utilization of noise reduction facilities were selected. These results were visualized using a forest plot (Figure 2). In the multiple-variable-adjusted logistic regression model, small and micro-sized enterprises were found to have a lower risk of noise exceedance compared to large and medium-sized enterprises (OR = 0.32, 95% CI: 0.16 to 0.64). Utilization of noise reduction facilities was also negatively associated with noise exceedance (OR = 0.5, 95% CI: 0.29 to 0.85). As for industry-specific associations, the risk of noise exceedance in the manufacturing of metal products and in the railway, shipping, aerospace, and other transportation equipment manufacturing sectors was significantly higher - by approximately 231% and 1,118%, respectively (OR = 3.31, 95% CI: 1.61 to 6.82; OR = 12.18, 95% CI: 1.40 to 105.63) - compared with enterprises in the other industries. In contrast, enterprises in the printing and reproduction of recorded media showed a decreased risk of noise exceedance (OR = 0.29, 95% CI: 0.13 to 0.67).

Table 4 indicates the proportion of earplugs provided and worn at different noise exposure levels. As noise exposure increases, the proportion of earplugs provided also rises (Z = −2.30, P_trend_ = 0.0212), which reaches 100% when the noise exceeds 85 dB(A). Nonetheless, the proportion of earplugs worn does not show a significant increase with higher noise exposure. Specifically, when noise ranges from 83.11 to 86.07 dB(A), only 74.42% of staff wear earplugs.

**Table 4:** Personal protection under different noise exposure levels - Beijing, 2023.

Different quantiles	Lower than *P_5_* (*n* = 14)	*P_5_*~ (*n* = 57)	*P_25_*~ (*n* = 72)	*P_50_*~ (*n* = 71)	*P_75_*~ (*n* = 43)	*P_90_*~ (*n* = 29)	Total (*n* = 286)	*P_trend_*
Noise exposure level [dB(A)]	Less than 72.55	72.55~	77.80~	80.67~	83.11~	86.07~		
Proportion of earplugs provided, %	92.86	96.49	98.61	100	100	100	98.60	*Z* = −2.30, *P_trend_* = 0.0212
Proportion of earplugs wearing, %	92.31	82.46	83.33	91.55	74.42	86.21	84.62	*Z* = 0.31, *P_trend_* = 0.7556

## 4 Discussion

The national workers’ health monitoring system of China comprises two components: the national surveillance system of occupational disease (22) and the workplace occupational diseases hazardous items monitoring system (23, 24). Surveillance of occupational hazards in the workplace has been conducted by the Chinese government for 4 years since 2019. Through the nationwide surveillance of occupational disease hazard factors in the workplace of employers, the Chinese government has acquired information on the current status of occupational disease hazards and assessed the impact of exposure to such hazards on the health of laborers in critical industries. This study mainly analyzes the noise monitoring results of industrial enterprises in Beijing in 2023.

The analysis of the relationship between noise exposure levels and different enterprise scales showed that large and medium enterprises had a higher proportion of noise exposure levels exceeding 85 dB(A) than small and micro-sized enterprises. This finding contradicts monitoring results observed nationwide and in other provinces and cities (24–26). This phenomenon is mainly because the main large and medium-sized enterprises in Beijing fall under the category of the manufacturing of motor vehicles. The production processes in these enterprises, such as stamping, welding, painting, and final assembly, involve a large number of machines and equipment with concentrated distribution, which increases the probability of collision between working parts, resulting in high noise exposure levels (27–29). Therefore, this study proposes the following recommendations: (i) the motor vehicle manufacturing sector optimize production processes, particularly at key control posts, and enhance automation and mechanization equipment; (ii) occupational health management in enterprises be strengthened to minimize noise exposure for workers in noisy positions; (iii) it is crucial to emphasize the use of noise-proof earplugs by workers is crucial, as it can effectively reduce the risk of hearing loss or noise-induced deafness. Moreover, the surveillance results showed that the proportion of noise exposure levels exceeding 85 dB(A) and the noise median in Hong Kong, Macao and Taiwan invested enterprises were much higher than in other enterprises. This finding is basically consistent with the surveillance of noise exposure levels in industrial enterprises in the Jiangsu Province in 2022 (30). The higher noise exposure levels in these enterprises are related to their insufficient attention and investment in the prevention and treatment of occupational diseases (30).

The surveillance results showed that the enterprises involved in noise exposure in Beijing are mainly distributed in District H and District J, followed by District L and District M, with the least in District A. This distribution pattern is related to Beijing’s urban spatial structure construction policy called “one core, one main, one auxiliary, two axes, multiple points, and one district” (31). The highest noise exposure levels were observed in the manufacturing of metal products, followed by the manufacturing of motor vehicles. These findings highlight the clustering of industries and their regional distribution in Beijing. Therefore, targeted supervision and management measures should be developed, focusing on manufacturing hubs such as Districts H and J, while enhancing noise prevention and control in the metal products and motor vehicle manufacturing sectors.

The surveillance results also revealed a negative correlation between the use of noise reduction facilities and the proportion of noise exposure levels exceeding 85 dB(A). In contrast, a positive correlation was observed between the provision of noise-proof earplugs and noise exposure levels. These results demonstrate the significant impact of using noise-reduction facilities or earplugs in reducing noise exposure levels (32). However, the survey results indicate that a relatively low percentage of workers wear earplugs, which may be due to a lack of occupational health knowledge and insufficient awareness of occupational disease risks. Some workers find wearing protective equipment to be uncomfortable or inconvenient, which affects work efficiency. Additionally, others may be unaware of how to properly wear and use such gear (33). Tikka et al. (34) conducted a review of 29 studies to assess the effectiveness of non-pharmaceutical interventions for the prevention of occupational noise exposure or hearing loss. These interventions were compared to no intervention or alternative interventions, including engineering controls, administrative controls, personal hearing protection devices, and hearing surveillance. This paper indicates that engineering interventions - such as purchasing new equipment, segregating noise sources, installing panels or curtains around sources, and training on the proper use of earplugs - significantly reduce noise exposure levels.

In this study, the median noise level was 81.1 dB(A), slightly lower than noise levels in manufacturing in other regions of China. According to relevant research data (35), China’s occupational exposure limits (OELs) for noise are established at 85 dB(A), in line with international standards adopted by several countries, including those in the European Union and Australia. This regulatory threshold is notably lower than the 90 dB(A) exposure limits implemented in occupational safety frameworks of the United States and India. A review assessing major noise sources and noise levels in chemical manufacturing plants highlighted that in the USA, industries such as printing and publishing, petroleum and coal products manufacturing, nonmetallic mineral product manufacturing, transportation equipment manufacturing, and furniture and related product manufacturing exhibited average noise levels from 82 to 94 dB(A). Additionally, in the UK, the textile and steel industries showed average noise levels between 82 to 100 dB(A). Industries with higher noise levels included spinning mills in Ethiopia and fluid crackers in the U.S. petroleum industry, where the average noise levels ranged from 86 to 115 dB(A) and 89 to 115 dB(A), respectively (36). In Saudi Arabia, noise levels in metalworking factories often exceeded 90 dB(A), while woodworking factories had slightly lower levels, though still significant, with 50% exceeding 85 dB(A) (37). A study evaluating the daily noise exposure of sawmill workers in Southwestern Nigeria found that noise levels ranged from 83.2 dB to 116.0 dB, with significant noise present during both idle and active work periods (38). Data from the Occupational Safety and Health Administration (OSHA) indicate that noise exposure levels across industries fluctuated between 1979 and 2013. In the manufacturing sector, the average noise level decreased from 90.8 dB(A) in 1979–1984 to 85.1 dB(A) in 2010–2013 (39). However, reducing noise levels alone is only a part of acoustic environment management, and improving the perception of the acoustic environment represents an important future direction. Recent research has shown that efforts to enhance the acoustic environment should probably be aimed at noise level reduction and human perception of soundscapes. For instance, W. Yang’ study found that the subjective evaluation of sound levels generally correlated with the mean L_eq_, especially when levels remained below 73 dBA (40). Notably, acoustic comfort is strongly influenced by sound source type - introducing pleasant sounds can considerably improve acoustic comfort, even at relatively high noise levels, possibly due to the psychological state of listeners (41). According to ISO, a soundscape is defined as an acoustic environment perceived, experienced and/or understood by individuals. Xu Zhang’s study demonstrated that the perceived dominance of certain sound sources significantly impacted relaxation, communication, spatiality and dynamics. Specifically, relaxation was greater when the natural sounds were dominant, while mechanical or anthropogenic sounds were associated with reduced relaxation. Acoustic comfort had a significant correlation with the soundscape dimensions and L_Aeq_ (42). Studies have found that ineffective noise control measures can lead to psychological annoyance. A study in a Brazilian public school found that teachers and students identified noise from adjacent classrooms and teachers’ voices as primary sources of disturbance (43). Besides, impulsive sounds, such as passing vehicles, tend to be perceived as more annoying than steady-state sounds with the same L_Aeq_ (44, 45). This difference in perceived noise annoyance can be accounted for by applying a penalty or an adjustment k to L_Aeq_ (45).

To mitigate noise hazards, it is crucial to strengthen occupational health supervision in industries, accelerate process reform, develop and deploy noise reduction technologies, and raise awareness of protection measures among workers. However, this study has several limitations: (i) some non-fixed-point operation posts or non-steady-state noise posts were not strictly monitored using individual measurement methods, which may have led to an underestimation of noise exceedance; (ii) crucial factors such as machine age, technological level, and working environment conditions were not recorded during surveillance. These factors, in addition to industry type and enterprise scale, may influence noise exceedance rates; and (iii) the present study did not collect hearing-related data from workers, and thus failed to analyze the relationship between noise exceedance and hearing loss. Future research could collect comprehensive data including machine age, technology level, and the hearing-related data of staff to optimize the logistic regression model and to assess the relationship between noise exposure and hearing loss.

## 5 Conclusion

In summary, the proportion of noise exposure levels exceeding 85 dB(A) in Beijing is 21.89%, lower than the national average of 25.14% (22). This difference may be attributed to Beijing’s industrial restructuring and reallocation of non-capital functions since 2014, a factor that should be further investigated. However, critical industries, especially the manufacturing of metal products and motor vehicles, still pose a significant noise exposure risk to workers.

Detailed surveillance of noise exposure levels is crucial for occupational health authorities to implement targeted supervision and develop specialized control measures for high-risk industries and job positions. Additionally, this data informs the formulation of the 14th Five-Year Plan for Occupational Disease Prevention and Control of Beijing City. To enhance noise monitoring efficiency, it is necessary to accelerate the adoption of intelligent occupational noise monitoring systems in workplaces. Recent research on intelligent noise monitoring has primarily focused on urban environmental noise. These methods, including acoustic cameras, machine learning (46–48) and Internet of Things technology (49–52), have significantly improved noise mapping accuracy. Furthermore, optimization techniques such as the CNOSSOS-EU model (53–55), neural network model (56), improved controlled passage method (CPB) (57), and integrated learning artificial intelligence (58–61) have enhanced environmental noise prediction. These methodologies offer implications for the intelligent monitoring of occupational noise in industrial enterprises. Future research should explore the use of machine learning and Internet of Things technology to realize real-time noise monitoring in workplaces, so as to reduce the cost of manual monitoring.

To safeguard employee privacy and uphold ethical compliance, the noise exposure monitoring process incorporated the following measures: (i) Data anonymization: monitoring data recorded only workstation codes and job categories, ensuring that personally identifiable information (e.g., employee names or personnel IDs) was not collected; (ii) Controlled monitoring scope: monitoring devices were deployed exclusively in designated work areas (e.g., workshops), explicitly excluding private spaces such as locker rooms and break rooms. Professional noise dosimeters, which lack audio recording capabilities, were used to eliminate the risk of voice interception and mitigate potential eavesdropping concerns; (iii) Data security protocols: raw data was encrypted and stored with tiered access controls, with scheduled deletion of temporary files to prevent traceability; (iv) (4) Informed consent: both enterprises and employees were informed in advance about the monitoring objectives and procedures; and (v) Regulatory adherence: the surveillance was conducted under the guidance of the Chinese Center for Disease Control and Prevention (China CDC) in strict compliance with the *Occupational Disease Prevention and Control Law* and so on.

These systematic measures ensured that the monitoring process maintained confidentiality and ethical integrity during noise exposure surveillance.

## Data Availability

The original contributions presented in the study are included in the article/Supplementary material, further inquiries can be directed to the corresponding authors.
