# Supplementary material for: Surveillance of noise exposure levels in workplaces in Beijing
Source: Front Public Health. 2025 Apr 29;13:1486497. doi: 10.3389/fpubh.2025.1486497 (PMC12071908; doi:10.3389/fpubh.2025.1486497)
Supplement: Supplementary file 1 [file Table_1.docx]

Supplementary Table 1. Variables in multivariate logistic regression model

| Variables | Code |
| --- | --- |
| Scale of enterprise | 1 = large and medium;  2 = small and micro-sized. |
| District | 1 = District A;  2 = District B;  3 = District C;  4 = District D;  5 = District E;  6 = District F;  7 = District G;  8 = District H;  9 = District I;  10 = District J;  11 = District K;  12 = District L;  13 = District M;  14 = District N;  15 = District O;  16 = District P. |
| Operating years | continuous variable |
| Participation of managers in occupational health training | 1 = yes; 0 = no. |
| Participation of occupational health managers in occupational health training | 1 = yes; 0 = no. |
| Provision of earplugs | 1 = yes (all the stuff wear earplugs); 0 = no (not all the stuff wear earplugs). |
| Posting of noise warning signs | 1 = yes; 0 = no. |
| Utilization of noise reduction devices | 1 = yes; 0 = no. |
| **Industries** |  |
| Electricity and heat production and supply industry | 1 = yes; 0 = no. |
| Manufacturing of other nonmetallic mineral products | 1 = yes; 0 = no. |
| Ferrous metal smelting and rolling industry | 1 = yes; 0 = no. |
| Manufacturing of chemicals and chemical products | 1 = yes; 0 = no. |
| Computer, communications and other electronic equipment manufacturing | 1 = yes; 0 = no. |
| Manufacturing of furniture | 1 = yes; 0 = no. |
| Manufacturing of metal products | 1 = yes; 0 = no. |
| Manufacturing of motor vehicles | 1 = yes; 0 = no. |
| Manufacturing of coke and refined petroleum products | 1 = yes; 0 = no. |
| Railway, shipping, aerospace and other transportation equipment manufacturing | 1 = yes; 0=no. |
| General equipment manufacturing | 1 = yes; 0 = no. |
| Pharmaceutical manufacturing industry | 1 = yes; 0 = no. |
| Instrumentation manufacturing industry | 1 = yes; 0 = no. |
| Printing and reproduction of recorded media industry | 1 = yes; 0 = no. |
| Nonferrous metal smelting and rolling industry | 1 = yes; 0 = no. |
| Special equipment manufacturing industry | 1 = yes; 0 = no. |
| Ownership type of enterprises | 1 = State-owned;  2 = Collective;  3 = Joint-equity cooperative enterprises;  4 = Joint-operate;  5 = Private;  6 = Incorporated company;  7 = Limited liability company;  8 = Hong Kong, Macau, and Taiwan invested enterprises;  9 = Foreign;  10 = Others. |
