# Supplementary figures and images for: Surveillance of noise exposure levels in workplaces in Beijing
Source: Front Public Health. 2025 Apr 29;13:1486497. doi: 10.3389/fpubh.2025.1486497 (PMC12071908; doi:10.3389/fpubh.2025.1486497)

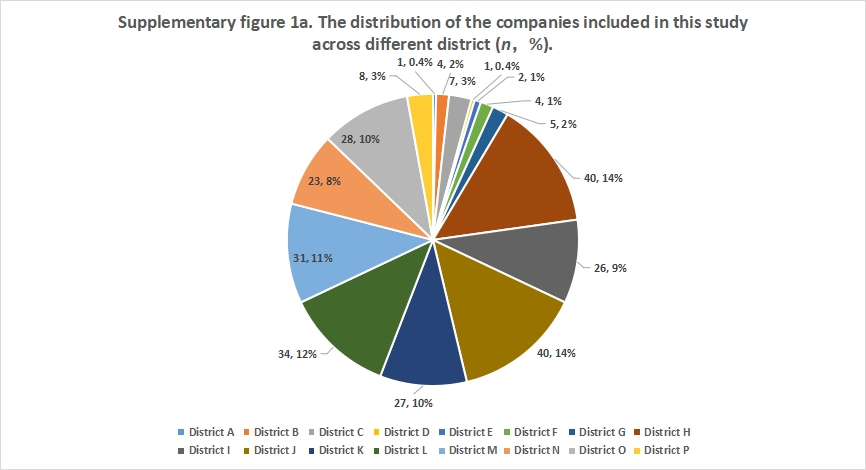


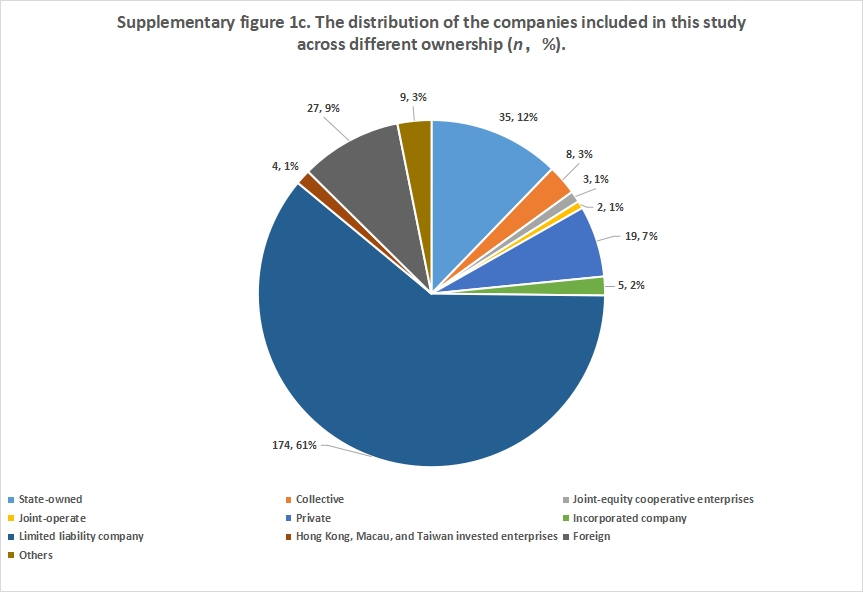

Supplement: Supplementary file 2 [file Data_Sheet_1.docx]
